# Chemical characterization of balloon flower (*Platycodon grandiflorum*) sprout extracts and their regulation of inflammatory activity in lipopolysaccharide‐stimulated RAW 264.7 murine macrophage cells

**DOI:** 10.1002/fsn3.1297

**Published:** 2019-12-03

**Authors:** Mina Kim, In‐Guk Hwang, Sang‐Bum Kim, Ae‐Jin Choi

**Affiliations:** ^1^ Division of Functional Food & Nutrition Department of Agrofood Resources National Institute of Agricultural Sciences Rural Development Administration Wanju Korea

**Keywords:** flavonoid, platycodi radix, polyphenol, root, saponin, sprout

## Abstract

The balloon flower (BF) is a potent natural source of phytochemical compounds and is associated with our health. The sprouting process is accompanied by significant changes in phytochemical compounds in comparison with their original plants. Even though many studies are conducted with BF, there are not yet reports of BF sprouts. In the present study, we determined the chemical composition and biological activity of BF sprouts that had been cultivated for 50 days. Kaempferol‐3‐O‐galactoside and 1‐O‐caffeoylquinic acid were identified as major components of whole BF sprouts. The leaves/stems of the sprouts had higher total phenolic and flavonoid contents and lower IC_50_ values in DPPH^•^ and ABTS^•+^ scavenging assays than whole sprouts or roots. The roots of the sprouts had the highest polygalacin D content (1.44 mg/g). We also determined the effects of different parts of BF sprouts on RAW 264.7 macrophage cells. When these cells were stimulated with lipopolysaccharide (LPS), their nitrite and pro‐inflammatory cytokine production increased. BF sprouts suppressed the LPS‐induced production of nitrite, tumor necrosis factor‐α, and interleukin‐6 in a concentration‐dependent manner without causing any cytotoxic effects. Nitrite and pro‐inflammatory cytokine production were significantly inhibited by the roots and leaves/stems, respectively. The inhibitory effects of BF sprouts on LPS‐stimulated inflammatory responses in RAW 264.7 macrophage cells were associated with suppressed NF‐κB activation. These findings suggest that BF sprouts could be a valuable source of bioactive compounds and exert anti‐inflammatory effects due to their polygalacin D, deapi‐platycodin D_3,_ and polyphenol content.

## Graphical abstract

Balloon flower sprouts could be a valuable source of bioactive compounds and exert anti‐inflammatory effects via suppression of NF‐kB activation due to their polygalacin D and polyphenol content.

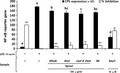

## 1 INTRODUCTION

Sprouted foods have recently come to be reviewed as functional foods and good sources of phytochemical compounds (Barillari et al., 2006; Limmongkon et al., 2018; Pham, David, & Barker, 2011). Many plants express high levels of antioxidants that play an important role in protecting themselves from oxidative stress during germination (Choi et al., 2013). The sprouting process is accompanied by significant changes in phenolic substances in comparison with their original plants (Kim, Park, & Lim, 2011). Antioxidant capacity, derived from phenolic compound content, significantly increased during germination in legumes (Lopez‐Amoros, Hernandez, & Estrella, 2006). Peanut sprout has higher resveratrol content than the ungerminated peanut kernel (Limmongkon et al., 2018).

Beneficial compounds from sprouts can be obtained not only from the roots but also from the leaves. Recent studies reported that leaves of ginseng sprout had higher ginsenoside contents than its roots (Kim et al., 2010; Park, 2012). Ginseng sprouts are considered a new medicinal vegetable that has begun to attract attention from consumers (Jang, Yu, Suh, Jang, & Kwon, 2018). Moreover, sprouts can be used after a relatively short period of germination and cultivation.

The balloon flower (BF, *Platycodon grandiflorum*) belongs to genus *Platycodon* L. of the family Campanulaceae (Jang et al., 2013). BFs are widespread in Northeast Asia, and BF roots (BRs, *Platycodi radix*, also called *doraji* in Korea and *jiegeng* in China) are commonly consumed as food and employed in traditional herbal medicine (Elijah, Jeong, Lee, & Jeong, 2014; Yoo et al., 2011). Certain saponins have been identified in BRs, including platycodins, polygalacin D, platyconic acid A, and platycosides (Park, Lee, Kim, Lee, & Kim, 2012). These various platycodin saponins have shown diverse pharmacological activities and strongly suppress inflammatory responses by blocking the generation of pro‐inflammatory mediators (Elijah et al., 2014; Park, Lee, et al., 2012). Choi et al., 2001 reported that 22‐year‐old BRs proved beneficial to adult patients with diabetes and lung cancer, and aqueous extracts from BRs that had been cultivated for more than 20 years inhibited the growth of tumors in mice. BRs are commonly harvested for their functional effects after quite a long duration (e.g., 4 years) of growth (Elijah et al., 2014; Park, Kim, Lee, Kim, & Baik, 2012).

Even though many studies are conducted with BRs, there are not yet reports describing the chemical composition and functionality of BF sprouts. Therefore, in this study, flavonoids from whole BF sprouts, and saponins from different parts of BF sprouts (i.e., whole sprouts, roots, and leaves/stems) were analyzed. We then studied the effects of BF sprout extracts in an in vitro model of inflammation and found that these extracts could ameliorate inflammatory factors by inhibiting nuclear factor kappa‐B (NF‐κB) activation and regulating cytokine production in RAW 264.7 macrophages.

## 2 MATERIALS AND METHODS

### 2.1 Chemicals and reagents

Folin‐Ciocalteu (F‐C) reagent, sodium carbonate (Na_2_CO_3_), gallic acid, aluminum trichloride (AlCl_3_), potassium acetate (CH_3_COOK), quercetin, 1,1‐diphenyl‐2‐picrylhydrazyl (DPPH), ascorbic acid, potassium persulfate (K_2_S_2_O_8_), 2,2’‐azino‐bis‐(3‐ethylbenzthiazoline‐6‐sulfonic acid) diammonium salt (ABTS), lipopolysaccharide (LPS), Griess reagent, and Bay 11–7082 were supplied by Sigma‐Aldrich Co. Galangin was obtained from Extrasynthese. Kaempferol‐3‐O‐galactoside, kaempferol‐3‐O‐neohesperidoside, 1‐O‐caffeoylquinic acid, apigenin‐7‐O‐glucoside, luteolin 7‐O‐(6''‐O‐malonyl)‐glucoside, 2,4,5‐trimethoxycinnamic acid, kaempferol‐3‐O‐(6‐O‐acetyl)B‐D‐glucopyranoside, kaempferol, apigenin‐7‐O‐B‐D‐glucuronide ethyl ester, and 1,5‐O‐dicaffeoylquinic acid were bought from Sigma. Deapi‐platycoside E, platycoside E, deapi‐platycodin D_3_, deapi‐platycodin D, platycodin D_2_, platycodin D, polygalacin D, and platyconic acid A were purchased from ChemFaces. Acetonitrile (ACN), ethanol (EtOH) and purified water were purchased from Fisher Scientific. Dulbecco's modified Eagle's medium (DMEM) was purchased from Lonza, fetal bovine serum (FBS) was obtained from Gibco, and penicillin–streptomycin (Pen/Strep) was bought from Hyclone. EZ‐Cytox was supplied by Daeilbio.

### 2.2 Preparation of balloon flower sprout extracts

BF sprouts were collected from a farm in Hoengseong‐gun, Gangwon Province, South Korea in 2018. BF seeds were germinated, and sprouts were cultivated for 50 days before being collected. In a total of 100 samples, the average size of a whole BF sprout was about 19 cm (leaf and stem, 11 cm; root, 8 cm) and the average weight was about 46 g. Two‐year‐old BRs (Etteum) were collected from a farm in Yeongdong‐gun, Chungbuk Province, South Korea in 2018. The BF sprouts and BRs were washed. The leaves (including the stems) were separated from the roots of BF sprouts. The raw sprouts and BRs were freeze‐dried (chamber pressure of 20 mTorr, shelf temperature of −40°C, cold trap temperature of −70°C; Bondiro, programmable freeze‐dryer, Ilshin Co. Ltd.), pulverized and stored at −70°C prior to LC analysis. For experiments other than LC analysis, 40 ml of 70% EtOH was added to 2 g of the freeze‐dried powder and subjected to 30 min of ultrasonic extraction for extract preparation. The extract was then centrifuged at 1,500 x *g* at 4°C for 10 min, concentrated by evaporation of the EtOH in a rotary vacuum evaporator (Eyela), freeze‐dried and stored at −70°C in an airtight polythene container.

### 2.3 Total phenolic content

The total phenolic content (TPC) was estimated by the F‐C method, as described by Elizabeth and Gillespie (Elizabeth & Gillespie, 2007), with slight modifications. Briefly, 100 μl of each extract in ethanol was mixed with 200 μl of 10% F‐C reagent and the mixture was kept at room temperature (RT) for 5 min. Then, 800 μl of 700 mM Na_2_CO_3_ was added to each tube. Subsequently, 200 μl of the mixture was transferred to 96‐well plate, and the absorbance was measured at 765 nm on a multimode microplate reader (Infinite M200 Pro; TECAN). The TPC was expressed as mg of gallic acid equivalents per g of freeze‐dried extract (GAE mg/g). All experiments were run in triplicate.

### 2.4 Total flavonoid content

The total flavonoid content (TFC) was determined by the AlCl_3_ method, and quercetin was used a reference compound (Anna & Andlauer, 2011). A volume of 100 μl of the ethanolic extract was mixed with 5 μl of 10% AlCl_3_ and 5 μl of a 1 M CH_3_COOK solution in a microplate. The final volume of the mixture was adjusted to 250 μl with distilled water. The mixture was allowed to stand for 30 min at RT. Then, the absorbance was measured at 415 nm. The TFC was expressed as mg of quercetin equivalents per g of freeze‐dried extract (QE mg/g). All experiments were run in triplicate.

### 2.5 Antioxidant activity

The free radical scavenging capacities of samples compared with ascorbic acid were determined by the DPPH^•^ and ABTS•^+^ scavenging assays. Extracts from different parts of the sprouts or from BRs were evaluated for their ability to scavenge DPPH radicals by the method of Blois (Blois, 1958) with suitable modifications. Briefly, 40 μl of ethanolic extracts at different concentrations were placed in the wells of a microplate. Subsequently, 160 μl of 0.15 mM DPPH in EtOH was added and the mixture was kept at RT for 30 min in the dark. The control was treated with 95% EtOH. A multimode microplate reader was used to measure of the absorbance at 517 nm.

For the production of ABTS^•+^, 7.4 mM ABTS was reacted with 2.6 mM K_2_S_2_O_8_ and stored in the dark at RT for 12 hr. The ABTS^•+^ solution was diluted to yield an absorbance of 1.2–1.5 at 734 nm. Then, 15 μl of ethanolic extracts at different concentrations were placed in the wells of a microplate. Subsequently, 285 μl of the ABTS^•+^ solution was added, and the absorbance was read at 734 nm after 5 min.

The percentage of scavenging was calculated from the obtained absorbance by the equation: % scavenging = ((Abs control‐Abs sample)/Abs control) × 100. Then, curves were constructed which the percentage of scavenging was plotted against the sample concentration in mg/ml (DPPH^•^) or µg/ml (ABTS^•+^). The equation of this curve was used to calculate the IC_50_, corresponding to the sample concentration that reduced the initial DPPH^•^ or ABTS^•+^ absorbance by 50%. A smaller IC_50_ value corresponds to higher antioxidant activity. All analyses were conducted in triplicate.

### 2.6 UPLC‐DAD‐QTOF/MS analysis

Each freeze‐dried powdered sample (0.1 g) was suspended in 10 ml of a 50% EtOH‐water solution (v/v) containing the internal standard (100 ppm of galangin and 2,4,5‐trimethoxycinnamic acid). The contents were sonicated at RT for 30 min in an ultrasonic bath (power, 300 W; frequency, 40 Hz). Then, the mixture was centrifuged at 1,500 x *g* at 4°C for 15 min. The supernatant was filtered through a 0.2‐µm PVDF syringe filter (Whatman, Kent). The analysis was performed on an ultra‐performance liquid chromatograph coupled with diode array detection and quadrupole time of flight mass spectrometry (UPLC‐DAD‐QTOF/MS; Waters Co.) equipped with a Cortecs UPLC T3 column (150 × 2.1 mm i.d., 1.6 μm; Waters Co.). Flavonoids were separated at a flow rate of 0.3 ml/min with mobile phases of ACN (A) and water (B). The following gradient conditions were used: 5% A (initial), 25% A (20 min), 50% A (25 min), 90% A (30–32 min), and 5% A (35–40 min). The chromatograms were acquired at 350 nm. Mass spectra were recorded in the range of *m*/*z* 200–1,200 with electrospray ionization as the ionization source in positive ion mode. The mass spectrometric setting was as follows: source temperature, 120°C; desolvation temperature 500°C; sampling cone voltage, 40 V; and desolvation N_2_ gas flow, 1,020 L/h. Instrument control, data acquisition and flavonoid and phenolic acid evaluations were performed with MassLynx V4.1 software (Waters Co).

### 2.7 HPLC‐UV/ELSD analysis

Each freeze‐dried powdered sample (0.1 g) was placed in 10 ml of 50% EtOH, and saponins were extracted in an ultrasonic bath at RT for 30 min as described above. Then, the extract was centrifuged at 1,500 x *g* for 10 min at 4°C. The residue was re‐extracted as mentioned above. The combined extract was completely evaporated and then diluted to 2 ml in a 50% EtOH‐water solution. High‐performance liquid chromatography‐ultraviolet analysis was performed, and the results were quantified with a light‐scattering detector (e2695, Waters Co). For this purpose, 20 μl of the filtrate was injected into the HPLC system. Compounds were separated on a Kinetex XB‐C_18_ column (250 mm × 4.6 mm, 5 μm, Phenomenex) with ultraviolet detection at 204 nm. The mobile phase was composed of ACN (A) and water (B) with gradient elution at a flow rate of 0.8 ml/min. The following linear gradient was applied: 10% A (0–5 min), 22% A (25 min), 28% A (50 min), 35% A (65 min), 60% A (70 min), 95% A (76–83 min), 50% A (89 min), and 10% A (94–105 min).

### 2.8 Cell culture and viability

RAW 264.7 mouse macrophage cells (TIB‐71, American Type Culture Collection) were maintained in DMEM supplemented with 10% FBS and 100 U/ml Pen/Strep at 37°C in a humidified 5% CO_2_ incubator. For all experiments, cells were grown to 80%–90% confluence and subjected to no more than 10 passages. Cells were seeded in 96‐well plate (5 × 10^5^ cells/well) and allowed to adhere. Then, the cells were treated for 24 hr with different concentrations of the samples (0–500 μg/ml); untreated cells were used as controls. The medium was replaced with fresh medium containing EZ‐Cytox solution (0.01 ml/well), and the cells were incubated at 37°C for 2 hr. The absorbance was then measured at 450 nm on a microplate reader. Cell viability (%) was calculated by the following equation: (absorbance of the sample/mean absorbance of the control) × 100.

### 2.9 Quantification of nitrite and cytokine production

The concentrations of nitrite, tumor necrosis factor‐α (TNF‐α), and interleukin‐6 (IL‐6) in the cell culture supernatants were measured with the Griess reagent for nitrite and with ELISA kits for TNF‐α and IL‐6 (Alpco). RAW 264.7 cells were plated in a 12‐well culture plate at a density of 2.4 × 10^6^ cells/well. The cells were pretreated with the samples for 2 hr prior to 24 hr treatment with 1 μg/ml LPS. The supernatants of cells cultured with or without the samples were then analyzed. For the measurement of nitrite levels (Yook, Kim, Kim, & Cha, 2017), equal volumes of the culture medium and Griess reagent were mixed and incubated at 37°C for 15 min. The resultant absorbance at 530 nm was measured on a microplate reader. The nitrite release of the treated cells was expressed as a percentage of that of untreated control cells. The concentrations of TNF‐α and IL‐6 were calculated from standard curves developed with known concentrations of recombinant TNF‐α and IL‐6 according to the manufacturer's protocols.

### 2.10 NF‐κB reporter gene assay

For the investigation of NF‐κB activation, RAW 264.7 cells stably transfected with an NF‐κB luciferase reporter were cultured by GBSA Bio center. The cells were seeded in 384‐well white plates (2 × 10^4^ cells/well) and allowed to adhere for 24 hr. Then, the cells were pretreated with the samples (100 μg/ml) or Bay11‐7082 (10 μM) for 30 min and were stimulated with LPS (10 ng/ml) for 3 hr. Untreated cells were used as controls, and Bay11‐7082 (an inhibitor of cytokine‐induced IκB‐α phosphorylation) was used for the positive control group. After that, 20 μg/ml Bright‐Glo (Bright‐Glo luciferase assay system, Promega) was applied to the cells through a JANUS MDT multiple pipetting system (Perkin‐Elmer, Inc.), and the cells were incubated for 2 min. Luciferase activation was measured with an EnVision Xcite Multilabel reader (Perkin‐Elmer, Inc., Ultrasensitive Luminescence mode).

### 2.11 Statistical analysis

All the data in this study were processed by SPSS (software version 20.0; IBM Corp.). Comparisons were made by one‐way analysis of variance (ANOVA) and Duncan's multiple range tests. An independent *t* test was used to examine nitrite and cytokines levels. A *p‐*value <.05 was considered to denote statistical significance.

## 3 RESULTS AND DISCUSSION

### 3.1 Antioxidant content and activity of balloon flower sprout extracts

Phenolic compounds including phenolic acids and flavonoids are secondary plant metabolites (Sadegh et al., 2014). Due to the antioxidant properties of these compounds, there is a growing interest in using them for disease prevention (Elijah et al., 2014). We found higher TPCs and TFCs in whole BF sprouts (83.37 GAE mg/g and 46.21 QE mg/g) than in BRs (22.33 GAE mg/g and 2.06 QE mg/g) (Table 1). The highest TPCs and TFCs were detected in leaves and stems (130.62 GAE mg/g and 101.76 QE mg/g). A previous study demonstrated that the aerial parts of BFs had strong antioxidant activity that correlated with high concentrations of phenolic compounds (Jeong et al., 2010). The relatively high levels of antioxidant compounds in the whole sprouts may reflect the high levels in the leaves and stems.

**Table 1:** Total phenolic content, flavonoid content, and radical scavenging IC_50_ values of extracts from different parts of balloon flower sprouts (*n* = 6)

	TPC (GAE mg/g FD extract wt.)	TFC (QE mg/g FD extract wt.)	DPPH^·^ IC_50_ (mg/ml)	ABTS^·+^ IC_50_ (μg/ml)
BF sprout				
Whole	83.37 ± 7.95^b^	46.21 ± 4.79^b^	0.738 ± 0.020^b^	3.100 ± 0.230^c^
Root	22.33 ± 6.42^c^	2.06 ± 0.24^c^	19.306 ± 6.485^a^	23.250 ± 2.943^b^
Leaf and stem	130.62 ± 16.13^a^	101.76 ± 7.31^a^	0.312 ± 0.007^b^	1.485 ± 0.124^d^
BR	16.90 ± 5.44^c^	0.47 ± 0.09^c^	21.360 ± 7.591^a^	31.149 ± 0.502^a^
AA	**–**	**–**	0.013 ± 0.000^b^	0.134 ± 0.005^d^
*F*‐value	355.839^***^	714.454^***^	36.214^***^	687.691^***^

Note

A different superscript letters in a column indicates a significant difference among groups by ANOVA with Duncan's multiple range test (^***^ *p* < .001).

Abbreviations: AA, ascorbic acid; BF, balloon flower; BR, balloon flower root; FD, freeze‐dried; GAE, gallic acid equivalents; QE, quercetin equivalents; TFC, total flavonoid content; TPC, total phenolic content; wt, weight.

**Figure 1:**
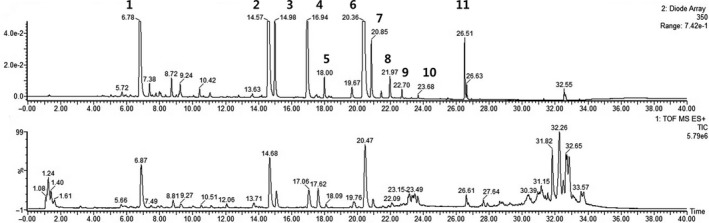
UPLC‐DAD‐QTOF/MS chromatograms of phenolic acids and flavonoids in balloon flower sprouts. (1), 1‐O‐caffeoylquinic acid; (2), Kaempferol‐3‐O‐galactoside; (3), Kaempferol‐3‐O‐neohesperidoside; (4), Apigenin‐7‐O‐glucoside; (5), Luteolin 7‐O‐(6''‐O‐malonyl)‐glucoside; (6), 2,4,5‐trimethoxycinnamic acid, internal standard; (7), Kaempferol‐3‐O‐(6‐O‐acetyl)B‐D‐glucopyranoside; (8), Kaempferol; (9), Apigenin‐7‐O‐B‐D‐glucuronide ethyl ester; (10), 1,5‐O‐Dicaffeoylquinic acid; and (11), Galangin, internal standard

The flavonoid composition of BF sprouts was analyzed by UPLC‐DAD‐QTOF/MS, and the results are shown in Table 2 and Figure 1. Seven kinds of flavonoids (derivatives of kaempferol, apigenin, and luteolin) and two kinds of phenolic acids (caffeoylquinic acid derivatives) were identified. Among them, kaempferol‐3‐O‐galactoside and 1‐O‐caffeoylquinic acid were found to be major compounds. We hypothesized that these compounds could confer antioxidant properties on BF sprout extracts. Takaya et al. reported that radish sprouts which contain kaempferol‐3‐O‐α‐L‐rhamnopyranosyl‐(1–4)‐β‐D‐glucopyranoside and kaempferol‐3,7‐O‐α‐L‐dirhamnopyranoside, exhibited the highest antioxidant activity among 11 commercially available vegetables in Japan (Takaya, Kondo, Furukawa, & Niwa, 2003). Kaempferol derivatives from an extract of *Capparis spinosa* displayed significant antioxidant activity in healthy humans, and kaempferol‐3‐O‐galactoside reduced the levels of hepatic lipid peroxidation and increased the levels of reduced glutathione in mice (Calderón‐Montaño, Burgos‐Morónv, Pérez‐Guerrero, & Lopez‐lazaro, 2011). Moreover, Jeong et al. found that the aerial parts of BFs contained high concentrations of apigenin‐7‐O‐glucoside and luteolin‐7‐O‐glucoside (Jeong et al., 2010). Another study suggested that the free radical scavenging activity of *Arctium lappa* could be attributed to caffeoylquinic acid derivatives (Maruta, Kawabata, & Niki, 1995). *Arctium lappa*, commonly known as burdock, is used for its root and has long been cultivated for food and folk medicine, similar to BFs (Predes, Ruiz, Carvalho, Foglio, & Dolder, 2011). Caffeoylquinic acids were recently identified as major constituents of the aerial parts of *Chrysanthemum coronarium* L. (Wan, Li, Liu, Chen, & Fan, 2017).

**Table 2:** Phenolic acids and flavonoids from balloon flower sprout

Peak number	Retention time (min)	Expected neutral mass	QTOF/MS (ESI) *m*/*z*	Fragment productions (*m*/*z*)	Identification
1	6.78	354.1	355.1	377, 355, 163	1‐O‐caffeoylquinic acid^a^
2	14.57	448.0	449.1	449, 287	Kaempferol−3‐O‐galactoside^a^
3	14.98	594.2	595.2	595, 287	Kaempferol−3‐O‐neohesperidoside
4	16.94	432.0	433.1	433, 271	Apigenin−7‐O‐glucoside
5	18.00	534.0	535.1	535, 287	Luteolin 7‐O‐(6''‐O‐malonyl)‐glucoside
6	20.36	238.2	239.2	239, 221	2,4,5‐trimethoxycinnamic acid (ISTD)
7	20.85	490.0	491.1	491, 287	Kaempferol−3‐O‐(6‐O‐acetyl)B‐D‐glucopyranoside
8	21.97	286.0	287.1	287	Kaempferol
9	22.70	474.1	475.1	621, 475, 271	Apigenin−7‐O‐B‐D‐glucuronide ethyl ester
10	23.68	516.1	517.3	517	1,5‐O‐Dicaffeoylquinic acid
11	26.51	270.0	271.1	271	Galangin (ISTD)

Abbreviations: ISTD, internal standard.

a Major components.

Considering the flavonoid and phenolic content of extracts from different parts of BF sprouts, along with the importance of flavonoids and phenolic acids as antioxidants in the human diet (Jeong et al., 2010), we evaluated the antioxidant activity of BF sprout extracts with DPPH^•^ and ABTS^•+^ scavenging assays. These results are shown as IC_50_ values in Table 1. Consistent with the results on the total antioxidant content, the IC_50_ values for scavenging DPPH and ABTS radicals were significantly lower in whole sprout extract (0.738 mg/ml and 3.1 μg/ml, respectively) than in BR extracts (21.36 mg/ml and 31.15 μg/ml, respectively). In a previous study, an IC_50_ values below 1 mg/ml for scavenging DPPH was considered to be a high antioxidant activity for a food, and white grapes had an IC_50_ value of 0.73 mg/ml (Qusti, Abo‐khatwa, & Lahwa, 2010). These results demonstrated the remarkable abilities of BF sprouts both to scavenge reactive oxygen species by donating hydrogen (DPPH^•^) and to scavenge proton radicals by donating electrons (ABTS^•+^) (Chu, Lim, Radhakrishnan, & Lim, 2010). The antioxidant capacity of BF sprout is attributed to the presence of polyphenolic compounds.

### 3.2 Saponin content of extracts from different parts of balloon flower sprouts

**Figure 2:**
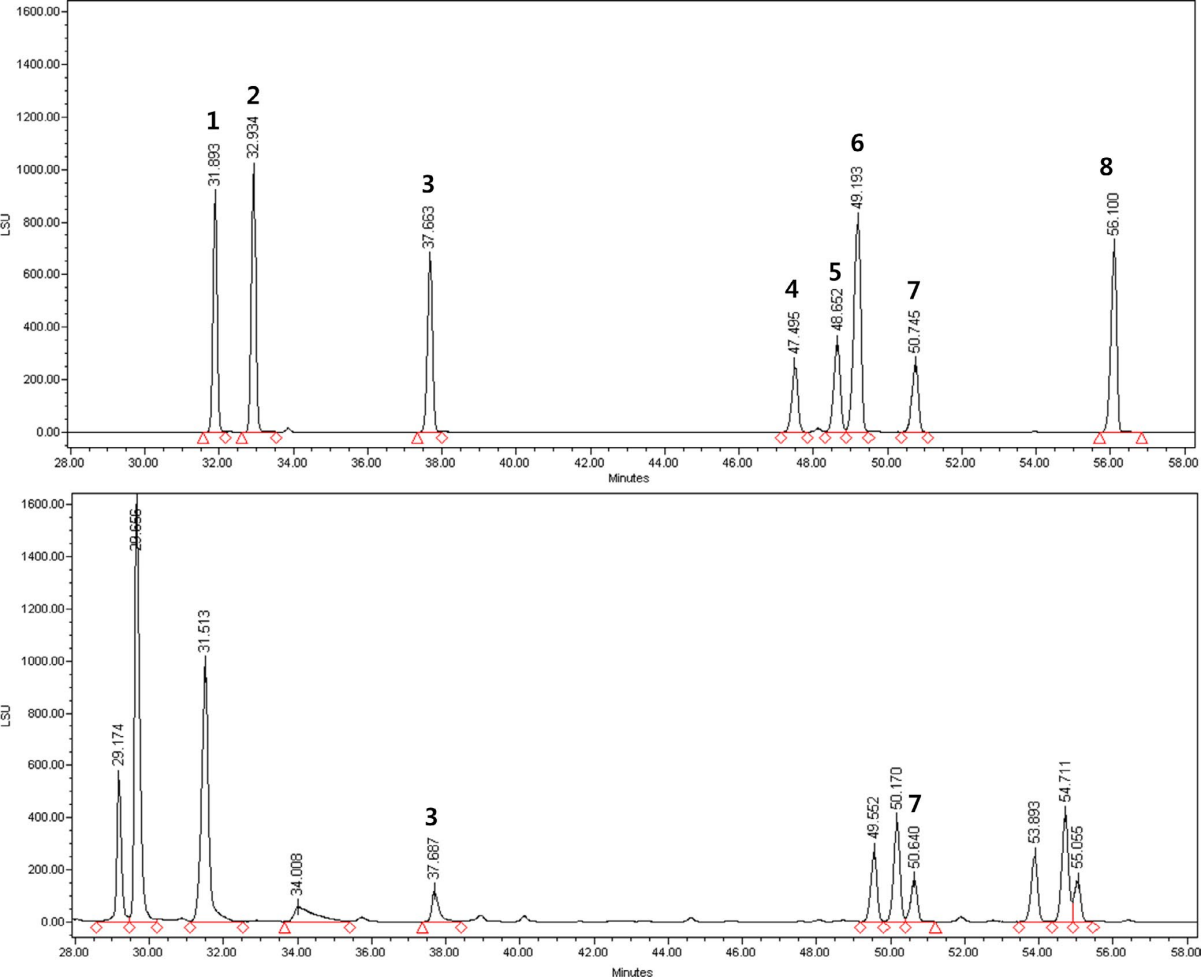
HPLC‐UV/ELSD chromatograms of saponins in balloon flower sprouts. (1), Deapi‐platycoside E; (2), Platycoside E; (3), Deapi‐platycodin D_3_; (4), Deapi‐platycodin D; (5), Platycodin D_2_; (6), Platycodin D; (7), Polygalacin D; and (8), Platyconic acid A

BRs are known for their high saponin and triterpenoid contents (Lee, Al‐Dhabi, Yan, Arasu, & Park, 2016). Saponins are glycosidic compounds present in various inedible and edible plants (Ahn et al., 2005). To date, more than 55 triterpenoid saponins have been isolated from BRs (Natalia, Joanna, Olga, & Szypuła, 2014). In one study (Elijah et al., 2014), ten major triterpenoid saponins were identified in BRs, including platycoside E, deapi‐platycoside E, platycodin A, platycodin D_3_, deapi‐platycodin D_3_, polygalacin D, 2''‐O‐acetyl polygalacin D, and 3''‐O‐acetyl polygalacin D. Yoo et al., 2011 reported that, among analyzed platycosides in 3‐year‐old BRs, platycoside E was the most abundant (2.00 mg/g), followed by polygalacin D_2_ (1.77 mg/g) and 3"‐O‐acetylplatyconic acid A (1.35 mg/g). Various platycodon saponins from BRs are known to exhibit anti‐inflammatory activity by blocking the generation of pro‐inflammatory mediators (Elijah et al., 2014; Park, Lee, et al., 2012). In this study, we analyzed different parts of BF sprouts (including whole sprouts) for eight kinds of saponins, but interestingly, only polygalacin D and deapi‐platycodin D_3_ were detected (Figure 2). The polygalacin D content was the highest in the roots (roots 1.44 ± 0.02 mg/g, whole sprouts 1.15 ± 0.01 mg/g, leaves/stems 0.97 ± 0.01 mg/g). On the other hand, the deapi‐platycodin D_3_ was the highest in the leaves/stems (roots 0.37 ± 0.00 mg/g, whole sprouts 0.58 ± 0.03 mg/g, leaves/stems 0.88 ± 0.04 mg/g).

Ahn et al., 2005 investigated various saponins and found that polygalacin D was a potential inhibitor of LPS‐induced nitrite production. The authors suggested that the single glucosyl group in the R1 position of the polygalacin D triterpenoid saponins is responsible for their anti‐inflammatory properties (Ahn et al., 2005). In addition, phenolic compounds and flavonoids are well known as antioxidants with immune‐system‐promoting and anti‐inflammatory effects (Tungmunnithum, Thongboonyou, Pholboon, & Yangsabai, 2018). Therefore, we hypothesized that BF sprouts could down‐regulate inflammation due to their flavonoid and polygalacin D content.

### 3.3 BF sprouts reduce inflammation‐related factors in LPS‐stimulated RAW 264.7 cells

Certain dietary components are thought to ameliorate low‐grade inflammation that can eventually result in chronic diseases such as cardiovascular disease, cancer, and Alzheimer's disease (Kong, Lee, & Wei, 2019). Thus, there is widespread interest in identifying additional dietary compound with anti‐inflammatory activity. BRs have been widely used in medicine as folk remedies for chronic inflammatory diseases (Ahn et al., 2005). To the best of our knowledge, this is the first study to evaluate the anti‐inflammatory properties of BF sprouts. As a first step, we conducted a cytotoxicity assay to evaluate the effects of BF sprout extracts on the viability of RAW 264.7 cells (Table 3). Concentrations of BF sprout extracts and BR extracts from 5 to 400 μg/ml were regarded to be in an appropriate range for further studies, as they did not exhibit any cytotoxic effects in RAW 264.7 cells (cell viabilities were over 94% relative to the untreated control group).

**Table 3:** Effects of extracts from different parts of balloon flower sprouts on cell viability in RAW 264.7 macrophages

μg/ml	% Cell viability			
	BF sprout			BR
	Whole	Root	Leaf and stem	
0	100.00 ± 3.21^e^	100.00 ± 5.38^de^	100.00 ± 9.86^d^	100.00 ± 15.28^c^
5	95.74 ± 4.54^e^	104.73 ± 5.68^d^	99.78 ± 3.82^d^	101.96 ± 5.78^c^
25	100.21 ± 9.00^e^	116.00 ± 7.56^c^	96.21 ± 5.03^d^	111.54 ± 12.49^c^
50	112.85 ± 5.49^d^	122.86 ± 12.43^c^	113.27 ± 9.05^c^	133.19 ± 8.19^b^
100	136.18 ± 7.50^b^	139.94 ± 12.48^b^	133.15 ± 20.36^b^	129.30 ± 13.20^b^
200	152.01 ± 8.81^a^	154.36 ± 9.60^a^	142.02 ± 12.58^b^	139.05 ± 10.06^b^
400	151.63 ± 6.70^a^	93.84 ± 8.79^e^	162.73 ± 7.94^a^	131.03 ± 16.44^b^
500	123.79 ± 8.20^c^	64.90 ± 4.88^f^	128.84 ± 8.02^b^	162.78 ± 10.53^a^
*F*‐value	54.68793^***^	60.13967^***^	28.93144^***^	18.38652^***^

Note

Cell viability was measured by cytotoxicity assays and calculated relative to the untreated control value (0 μg/ml). Data are expressed as means ± SDs. Means with different superscripts in the same column differ significant (^***^ *p* < .001).

Abbreviations: BF, balloon flower; BR, balloon flower root.

**Figure 3:**
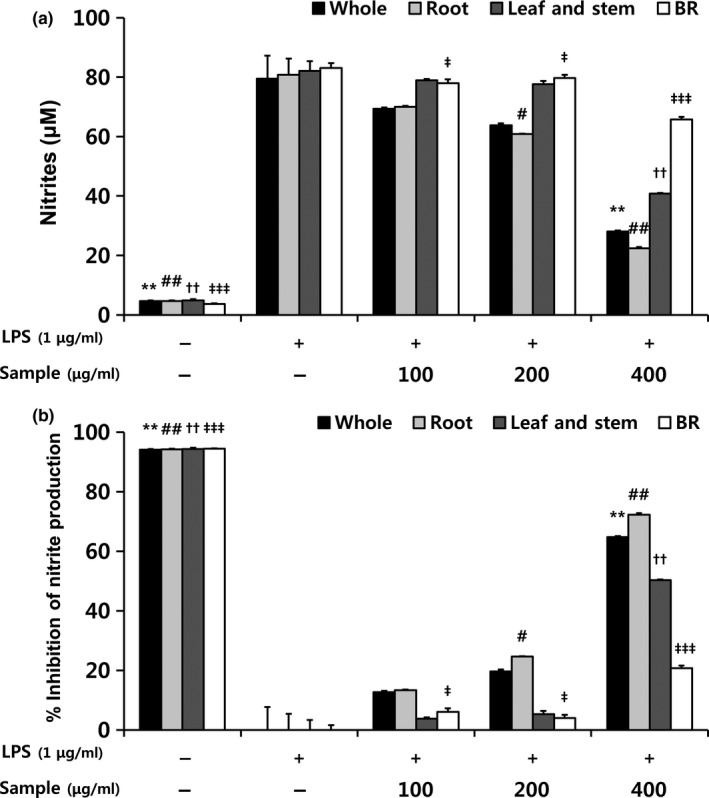
Effects of extracts from different parts of balloon flower sprouts on (a) the nitrite concentration and (b) the %inhibition of nitrite production in RAW 264.7 macrophages. BR, balloon flower root. Data are expressed as means ± SDs. The LPS‐stimulated group without sample treatment was compared with each indicated group by an independent *t* test (^*, #, †, ‡^ *p* < .05, ^**, ##, ††, ‡‡^ *p* < .01, and ^***, ###, †††, ‡‡‡^ *p* < .001). *, whole sprouts; #, roots of sprouts; †, leaves and stems of sprouts; ‡, BRs

**Figure 4:**
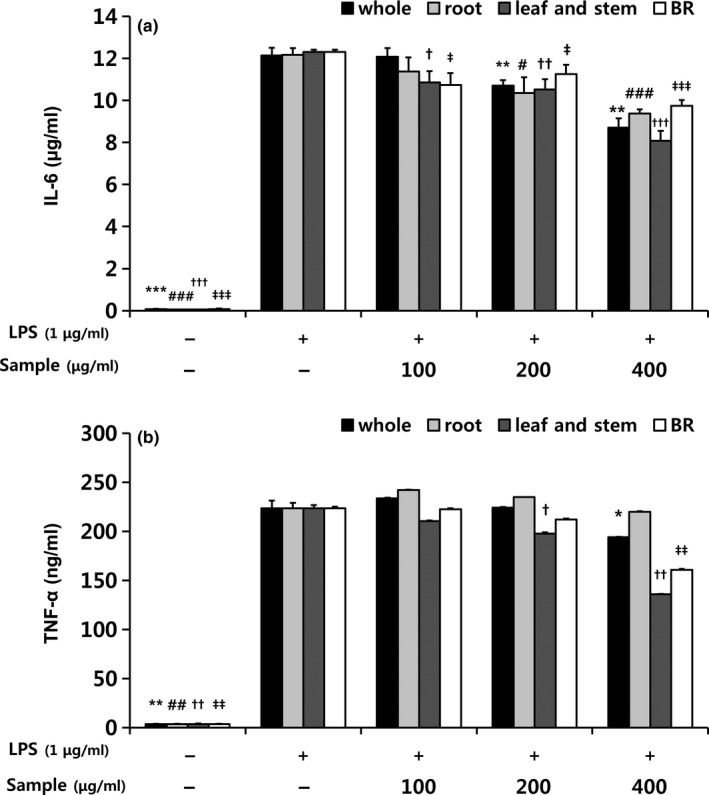
Effects of extracts from different parts of balloon flower sprouts on (a) TNF‐α and (b) IL‐6 concentrations in RAW 264.7 macrophages. BR, balloon flower root. Data are expressed as means ± SDs. The LPS‐stimulated group without sample treatment was compared with each indicated groups by independent *t* test (^*, #, †, ‡^ *p* < .05, ^**, ##, ††, ‡‡^ *p* < .01, and ^***, ###, †††, ‡‡‡^ *p* < .001). *, whole sprouts; #, roots of sprouts; †, leaves and stems of sprouts; ‡, BRs

To examine the anti‐inflammatory activity of BF sprouts, we stimulated RAW 264.7 cells with LPS and then treated them with varying extract concentrations. We then measured the extracellular levels of nitrite (Figure 3) and pro‐inflammatory cytokines such as TNF‐α and IL‐6 (Figure 4). The most prominent phenomenon in the process of inflammation is the increase in nitrite production (Jung, Kim, Park, Jeong, & Ko, 2018). Nitrite overproduction is one of the early signs of acute or chronic inflammation, and nitrite is released by various immune cells, including macrophages (Delgerzul, Muhammad, Im, Nisar, & Hwang, 2018). As shown in Figure 3a, LPS treatment triggered significant nitrite production, but this increase was effectively and concentration‐dependently inhibited by extracts from different parts of BF sprouts and BRs. BF sprout root extracts and whole sprout extracts inhibited nitrite production to the greatest extent relative to the LPS‐treated control (72% and 65%, respectively, at 400 μg/ml, Figure 3b).

**Figure 5:**
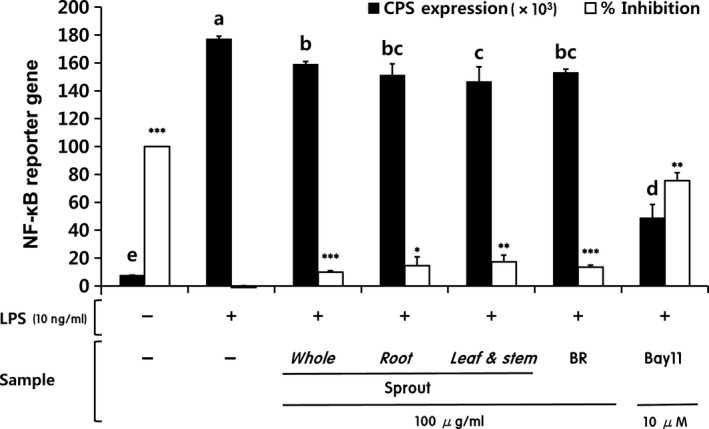
Inhibition of NF‐κB activity by extracts from different parts of balloon flower sprouts. BR, balloon flower root; Bay11, Bay11‐7082 (an inhibitor of cytokine‐induced IκB‐α phosphorylation). Data are expressed as means ± SDs. Different letters represent significant differences between groups according to one‐way analysis of variance followed by Duncan's multiple range test (*p* < .001). The LPS‐stimulated group without sample treatment was compared with each indicated group by independent *t* test (^*^ *p* < .05, ^**^ *p* < .01 and ^***^ *p* < .001)

LPS substantially induces macrophages to release pro‐inflammatory cytokines that upregulate inflammatory reactions (Delgerzul et al., 2018). BF sprout extracts significantly inhibited the LPS‐induced release of TNF‐ α and IL‐6 (Figure 4a,b). Leaf and stem extract particularly reduced TNF‐α and IL‐6 levels, and whole sprout extracts also significantly reduced IL‐6 levels (Figure 4b). IL‐6 is produced by macrophages at inflammatory sites (Delgerzul et al., 2018). Compared with other cytokines released during the inflammatory response, IL‐6 is a key stimulator of the acute inflammatory response (Gabay, 2006). TNF‐α plays various roles in inflammation and the immune system (Zelova & Hosek, 2013) and is known to induce the production of primary pro‐inflammatory mediators such as nitrite (Ren & Torres, 2009). TNF‐α is also known to activate NF‐κB, a transcriptional activator of NOD‐like receptor P3 (Liu, Zhang, Joo, & Sun, 2017). NF‐κB is a key transcription factor in macrophages and is required for the induction of a large number of inflammatory genes, including TNF‐α, IL‐1β, and IL‐6, which are ultimately secreted as pro‐inflammatory cytokines (Liu et al., 2017). The NF‐κB reporter gene was upregulated in the LPS group compared with the untreated control group (Figure 5). Treatment with Bay11‐7082, which inhibits the activation of NF‐κB by blocking the degradation of IκB‐α, significantly inhibited NF‐κB reporter gene expression in LPS‐stimulated cells. BF sprout extracts also significantly downregulated the NF‐κB reporter gene compared with the LPS group. Thus, BF sprout extracts may have suppressed the production of nitrite and cytokines by inhibiting NF‐κB activation. Other researchers have reported that various platycodin saponins inhibited pro‐inflammatory gene expression by blocking NF‐κB activation in LPS‐induced RAW 264.7 cells (Jang et al., 2013; Park, Lee, et al., 2012). A recent study suggested that NF‐κB was modulated by the interaction of polyphenols, phenolic acids, saponins, and triterpenoids (Serafini & Peluso, 2016).

According to these results, BF sprouts, which contain flavonoids and polygalacin D in their roots, leaves and stems, should be highly regarded for regulating inflammatory progression. However, further studies are required to elucidate the molecular mechanisms underlying the anti‐inflammatory effects of BF sprouts.

## 4 CONCLUSIONS

BRs have been used as food and employed in traditional herbal medicine, but years of growth are commonly needed to exploit their functional effects. On the other hand, sprouts are relatively easy to use because they can be cultivated in a short period of time, and beneficial compounds can be obtained both their roots and their leaves. However, research on BF sprouts has not yet been reported. Therefore, the results of this work are noteworthy, not only in demonstrating that BF sprouts exert antioxidant activity, but also in revealing that they contain a variety of phenolic acids and flavonoids. The antioxidant activity of BF sprout extracts was attributed to these phenolic compounds, particularly kaempferol‐3‐O‐galactoside and 1‐O‐caffeoylquinic acid. Furthermore, the inhibitory effects of BF sprout extracts on LPS‐stimulated inflammatory responses in RAW 264.7 macrophage cells were associated with the suppression of NF‐κB activation. BF sprout extracts inhibited the production of nitrite, TNF‐α, and IL‐6 without causing any cytotoxic effects in macrophage cells. Consequently, our results indicated that BF sprouts are a valuable and effective source of bioactive compounds and may regulate inflammatory activity due to their polygalacin D and polyphenolic content.

## CONFLICTS OF INTEREST

The authors declare no conflict of interest.

## ETHICAL APPROVAL

Neither animal nor human testing was involved in this study.
